# Comprehensive Evaluation of a Passive Upper-Limb Exoskeleton in Agricultural Tasks: Integrating Physiological, Postural, and Cognitive Measures

**DOI:** 10.3390/s25247640

**Published:** 2025-12-16

**Authors:** Sung-Seok Ko, Byungkyu Choi, Jaehyun Park, Mintae Seo, Jaejin Hwang

**Affiliations:** 1 Department of Industrial Engineering, Konkuk University, Seoul 05029, Republic of Korea; ssko@konkuk.ac.kr (S.-S.K.); bkchoi@konkuk.ac.kr (B.C.); 2 Rural Development Administration, National Institute of Agricultural Sciences, Jeonju-si 54875, Jeollabuk-do, Republic of Korea; mtseo85@korea.kr; 3 Department of Industrial and Systems Engineering, Northern Illinois University, DeKalb, IL 60115, USA; jhwang3@niu.edu

**Keywords:** upper-limb exoskeleton, muscle activity, perceived exertion, agricultural exoskeleton usability evaluation questionnaire (AUEQ)

## Abstract

This study evaluated the effects of a passive upper-limb exoskeleton in agricultural work using a repeated-measures design with 24 adult males across three exoskeleton conditions (No Exo, Prototype, and Airframe), three work directions (left, front, and right), and two work distances (near and far). Outcomes included muscle activity, center of pressure travel, task completion time, perceived exertion, body part discomfort, and usability. Airframe use significantly reduced shoulder and upper-arm muscle activity by about 25–35 percent, consistent with the mechanical sharing of shoulder elevation torque. Erector spinae activity showed a compensatory increase trend, suggesting heightened trunk stabilization demands. Center of pressure varied by direction and distance, with greater excursion for leftward and far tasks, but did not differ among exoskeleton conditions, indicating preserved postural stability. Task time was unaffected by exoskeleton and distance but was longest for leftward tasks. Perceived exertion and discomfort varied by direction and distance and tended to be lower on average with Airframe. Usability differed only in the effectiveness factor, which favored Airframe. An integrated evaluation emphasizing EMG, center of pressure, and perceived fatigue, with usability as needed, is recommended for field validation.

## 1. Introduction

Musculoskeletal disorders remain the most frequently reported occupational illnesses in industrial and agricultural settings [1,2,3]. These disorders mainly arise from repetitive upper-limb movements, prolonged non-neutral postures, and sustained muscular loads [4]. In particular, agricultural workers repeatedly maintain trunk flexion or elevate the upper limbs, which accumulates substantial mechanical burden on the shoulder and lower back regions [2]. This results in reduced productivity as well as long-term musculoskeletal damage, and is cited as a major health concern among agricultural workers.

As a technological approach to mitigating such physical burdens, the use of wearable assistive devices has attracted attention [5]. Exoskeletons are devices that mechanically assist specific joint motions of the human body to reduce muscular load and are classified by actuation into active (powered) and passive (unpowered) systems [6]. Active devices allow precise control but are heavy and power-hungry, which hinders field deployment [7], whereas passive devices feature lightweight structures and high energy efficiency, making them more feasible in real work environments [8,9]. However, studies verifying the effects of exoskeletons have concentrated mainly on electromyography (EMG) [10,11,12,13], and there is still insufficient evidence directly linking those results to cognitive and psychological indicators such as perceived fatigue (RPE) or usability.

Notably, prior studies report decreased muscle activity when wearing exoskeletons [14,15,16,17,18], but it remains unclear whether such physiological changes actually lead to improvements in task performance or maintenance of postural stability. Although indicators such as the center of pressure (CoP) can evaluate body stability during real tasks, they have been relatively overlooked in agricultural studies. Therefore, it is difficult to generalize exoskeleton effects based on a single physiological indicator; it is necessary to consider physiological, behavioral, and cognitive indicators in an integrated manner.

Accordingly, this study sought to clarify, from multiple angles, the effectiveness of an upper-limb assist exoskeleton in agricultural work environments. The commercially available Airframe and a Prototype designed by the research team were used as comparators, and two research objectives were established. First, we conducted an integrated analysis of changes in EMG, CoP, task efficiency (task completion time), subjective fatigue and discomfort (RPE/RPD), and the Agricultural Exoskeleton Usability Evaluation Questionnaire (AUEQ) according to exoskeleton use, work direction, and work distance in order to verify the comprehensive effects of wearing the devices on physical and cognitive burden. Second, we examined the correlations and representativeness among these multiple indicators and derived a combination of evaluation indicators that best explains exoskeleton effectiveness.

This study goes beyond evaluations of exoskeleton utility based solely on EMG reductions to investigate the linkage between mechanical assistance and human-centered experience. The aim is to provide an academic foundation for establishing evaluation systems and for the practical application of exoskeletons in agriculture.

## 2. Methods

### 2.1. Participants

This study included 24 healthy adult males with no history of musculoskeletal disorders and good overall health (mean age: 26.3 ± 3.1 years; height: 171.2 ± 7.8 cm; weight: 67.9 ± 9.4 kg). None had experienced pain or undergone surgery of the shoulder, back, or arms within the prior six months, and all were physically capable of normal daily activities. All participants were right-handed and were recruited only if they had no limitations in agricultural work or repetitive upper-limb activities.

Before the experiment, the purpose, procedures, device donning, and potential risks were fully explained verbally and in writing, and participants signed an informed consent form before taking part. All participants performed sufficient stretching and device acclimation before the main experiment. The study was approved by the institutional review board and complied with the ethical principles of the Declaration of Helsinki [19]. The researcher continuously observed participants to allow for immediate discontinuation upon any adverse signs (fatigue and discomfort), and rest was provided to all participants after the experiment.

### 2.2. Independent Variables

This study used a three-factor repeated-measures design with exoskeleton condition (No Exo, Prototype, and Airframe), work direction (left, front, and right), and work distance (near and far). The Prototype was an experimental device developed by the research team to assist the upper limbs during agricultural work, referring to the basic structure of the Airframe but improving joint alignment and donning convenience.

**Figure 1:**
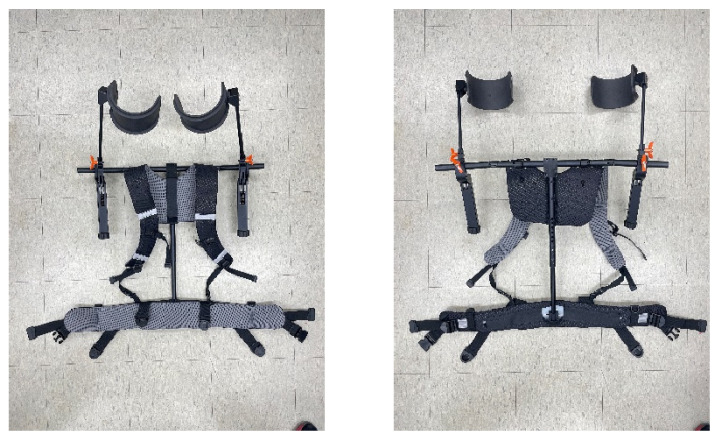
The developed Prototype exoskeleton.

The Airframe is a commercial product that shares shoulder elevation torque through a spring-based passive assist structure. The Airframe is a passive arm support device that employs a spring-driven assist mechanism and has a total mass of approximately 3.2 kg. For consistency across conditions and to isolate the effect of mechanical assistance, the assistive torque of the Airframe was standardized to 7.5 Nm during testing. The Prototype exoskeleton developed by our research team also adopts a spring-based passive assistance approach and has a weight similar to that of the Airframe. Its maximum spring output remains below 6 Nm at the highest tension setting (Figure 1). Structurally, the Prototype incorporates additional articulation to improve conformity with natural shoulder and arm motion, including two more joints than the Airframe, resulting in four articulated joints along the shoulder line—to enhance range-of-motion compatibility during agricultural tasks.

Note that the purpose of developing this Prototype was to create an exoskeleton specifically tailored for agricultural tasks. The research team designed and fabricated multiple custom components, including the spring modules, and integrated them into the system. The Prototype has continued to be refined and upgraded following the experiments. This Prototype represents an initial developmental version. Therefore, the present study was not intended to demonstrate superiority over existing commercial devices. Rather, we emphasize that the primary objective of this research was to establish and refine an evaluation protocol for agricultural exoskeletons.

Work direction (left, front, and right) and distance (near and far) were included because they are factors that greatly affect body balance and trunk load in actual agricultural work, and were added to evaluate how exoskeletons influence body center movement and balance maintenance strategies. Directional changes induce asymmetric posture (asymmetric posture) accompanied by trunk rotation and changes in the distribution of muscle activity, while distance changes adjust the lever arm and moment load to reproduce differences in upper-limb workload.

The order of conditions was randomized using a Latin square method to minimize order effects among participants, and a rest of two minutes was provided between conditions to prevent cumulative fatigue. An additional rest of approximately five minutes was provided between sessions to induce acclimatization and recovery.

### 2.3. Dependent Variables

To comprehensively evaluate the impact of wearing an exoskeleton on human load and task efficiency in agricultural work, five main dependent variables were set. Muscle activity was collected using a Noraxon wireless EMG system to quantitatively measure physiological load [20]. Signals were recorded from left and right upper trapezius (UT), anterior deltoid (AD), erector spinae (ES), and vastus lateralis (VL), and from the dominant (right) biceps brachii (BB). The sampling rate was 2000 Hz. Signals were processed through a 20–450 Hz band-pass filter and rectified, then normalized to percent maximum voluntary contraction (%MVC). Each muscle’s MVC was measured three times and averaged.

To assess postural stability, CoP data were obtained using a Nintendo Wii Balance Board (Nintendo Co., Ltd., Kyoto, Japan). The device incorporates four corner-mounted load cells that detect shifts in weight distribution, and its CoP measurements have been generally reported to achieve an accuracy on the order of approximately a few millimeters (commonly around 1–5 mm) in controlled laboratory environments. The board transmits digital force sensor outputs via Bluetooth, and all CoP signals were recorded at a sampling rate of 100 Hz using BrainBlox software (Neuromechanics Laboratory, University of Colorado Boulder, Boulder, CO, USA).

Task completion time was measured with a stopwatch from the start of each task to its end. The criterion movement was defined from the moment the arm was raised to reach the object to the moment the participant returned to the original posture.

Subjective fatigue (RPE) and body part discomfort (RPD) were used to evaluate perceived load immediately after each task. Whole-body fatigue was rated with Borg’s 6–20 scale, and discomfort was rated on a 0–10 scale [21]. RPD responses were obtained for nine body regions: neck, shoulder, upper back, elbow, wrist/hand, lower back, hip/thigh, knee, and ankle/foot.

Finally, usability of the exoskeletons was evaluated using the AUEQ [22]. The AUEQ is a validated subjective usability and comfort assessment tool for evaluating upper-limb exoskeletons. The AUEQ consists of 24 items rated on a 0–10 scale, and includes four sub-factors: Effectiveness, Wearability, Safety, and Learnability. Each factor reflects the actual assistive effect of the exoskeleton, donning comfort, safety perception, and ease of learning, respectively. This scale is a validated usability assessment tool for agricultural exoskeletons and was used to evaluate the consistency between user cognitive acceptance and actual efficiency.

### 2.4. Experimental Task

**Figure 2:**
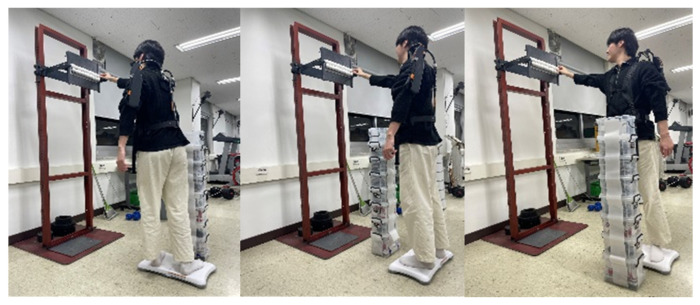
The harvesting task while the participant maintains a fixed stance on the balance board.

The experiment was organized around a harvesting motion that simulated a real agricultural work environment. All participants were controlled to use only their dominant (right) hand, and the positions and orientations of both feet were fixed to maintain consistency of movement. The stance width was maintained at 30 cm, and foot movement was not permitted during the experiment. The height of the work surface was set to 175 cm—corresponding to the participant’s shoulder height—to reproduce agricultural situations requiring forward elevation and extension of the upper limb. The harvest target was placed in a basket located directly in front of the participant, and the participant performed a sequence of movements: grasping the target object, lifting it, placing it into the basket, and then returning to the initial posture (Figure 2).

Tasks were presented by combining two factors, work direction (left, front, and right) and work distance (near and far). Work direction consisted of three levels based on the participant’s body midline—approximately 90° to the left, 0° to the front, and approximately 90° to the right—and was designed to evaluate changes in trunk rotation and the distribution of muscle activity due to asymmetric posture (asymmetric posture) of the upper limbs. Work distance was defined as the forward distance from the center of the work surface and set to two conditions: near (65.5 cm) and far (73.0 cm). These settings were intended to reproduce differences in upper-limb moment load and postural stability depending on the radius of reach. This experimental configuration was adapted with reference to the research protocol developed by Choi and Park (2025) [23].

Each condition was presented in a randomized Latin square order, with two minutes of rest between conditions to minimize cumulative fatigue. After sufficiently learning the procedures before the experiment, participants practiced in each condition and then participated in the main experiment. All experiments were conducted under researcher observation so that the participant’s posture, exoskeleton fit, and task performance were standardized.

### 2.5. Data Analysis

All experimental data were statistically analyzed to test the effects and interactions among factors. A multivariate analysis of variance (MANOVA) reflecting the repeated-measures design of 3 (exoskeleton condition: No Exo, Prototype, and Airframe) × 3 (work direction: left, front, and right) × 2 (work distance: near and far) was used. This method simultaneously considers multiple physiological, behavioral, and cognitive dependent variables to comprehensively verify the effects of exoskeleton wearing on overall task responses.

When MANOVA indicated significant main or interaction effects, univariate ANOVAs were performed for each dependent variable to confirm factor-specific effects. When ANOVA results were significant, the Student–Newman–Keuls (SNK) post hoc test was conducted to compare differences among conditions [24].

In addition, when significant interaction terms appeared, simple effect tests were conducted within levels of the other factors to examine in detail how exoskeleton effects changed under specific direction or distance conditions. The significance level (α) for all statistical tests was set at 0.05 [25], and data analysis was performed using SPSS (Version 28, IBM Corp., Armonk, NY, USA). Through these analytical procedures, the main effects and factor interactions of exoskeleton wearing on EMG, CoP, task completion time, RPE/RPD, and AUEQ were systematically verified.

## 3. Results

### 3.1. Analysis of Changes in Muscle Load

**Figure 3:**
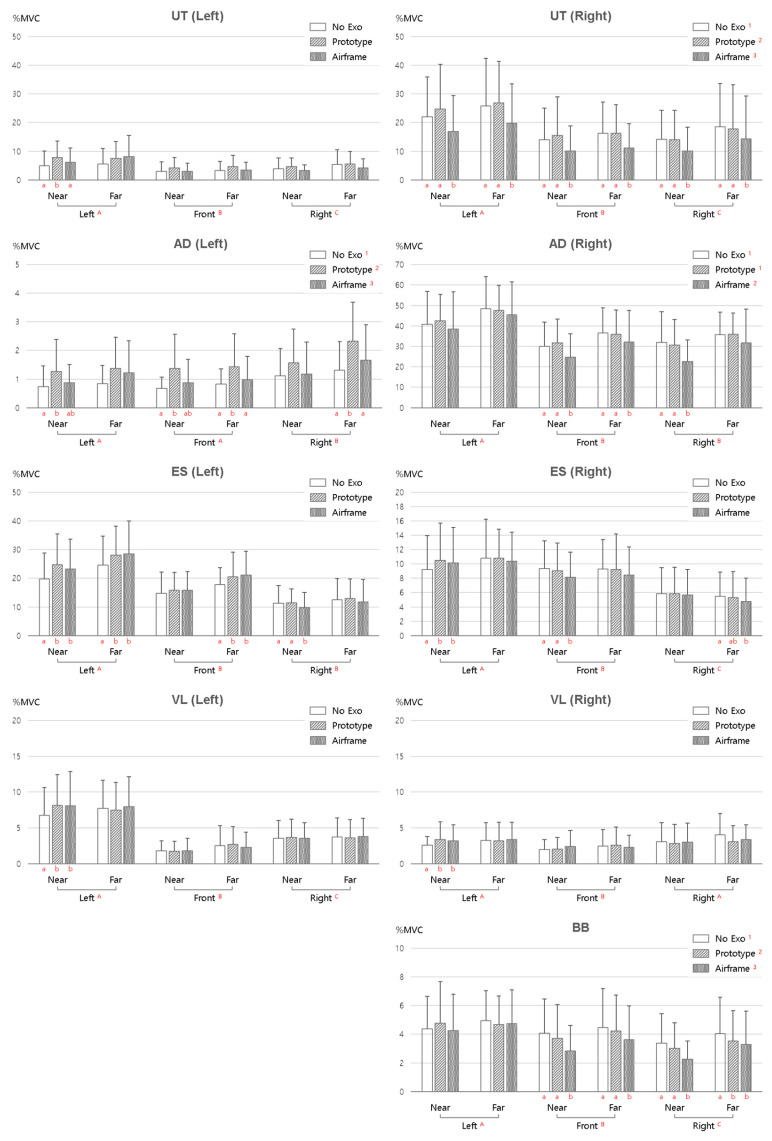
Normalized EMG (%MVC) across muscles by exoskeleton condition, work direction (left/front/right), and distance (near/far). (Capital letters A, B, and C represent statistically homogeneous groups identified by the SNK post hoc test and do not correspond to task directions. Superscript numerals ^1^, ^2^, and ^3^ denote significant differences among exoskeleton conditions, and lowercase letters a and b indicate significant simple-effect results).

Figure 3 presents mean normalized muscle activity (%MVC) according to three factors: exoskeleton condition (No Exo, Prototype, and Airframe), work direction (left, front, and right), and work distance (near and far). All post hoc comparisons used the SNK method. MANOVA results showed significant differences by exoskeleton condition for the right UT, left AD, right AD, and BB (*p* < 0.05). In these muscles, %MVC was significantly lower with Airframe than with No Exo or Prototype. Although the effects varied by muscle and task, the Airframe reduced muscle activity by up to approximately 32.7% in the BB compared with the no-exoskeleton condition, while the Prototype achieved reductions of up to approximately 23.7% MVC in the VL. However, some muscles exhibited increased activation depending on the task and posture. This is interpreted as the device’s spring structure sharing part of the torque during shoulder elevation, thus reducing the muscle contraction demands of the shoulder and arm. In contrast, the Prototype showed a tendency for increased muscle activity in certain direction–distance combinations, which is judged to be the result of compensatory muscle contractions due to subtle misalignments between the device’s assist axes and the body’s joint centers.

The erector spinae did not show statistically significant differences, but muscle activity tended to be lowest with No Exo and slightly higher when wearing Prototype or Airframe. This is interpreted as compensatory core activation to maintain stabilization of the trunk when exoskeleton wear reduces upper-limb load. That is, as load distribution by the exoskeleton requires balance control of the body center, trunk muscles may be additionally recruited.

The work direction factor showed statistically significant main effects for all muscles (*p* < 0.01). Generally, higher muscle activity was observed on the side of the body corresponding to the target direction; for example, during rightward tasks, %MVC in the right upper trapezius and anterior deltoid was highest. This shows that work direction is a key factor determining the distribution of muscle load in agricultural work, and suggests that torque distribution and degrees of freedom by direction should be considered in exoskeleton design. In addition, some simple effect test results showed that the Airframe’s effect of reducing muscle activity was prominent only in certain directions, indicating that the assist efficiency of exoskeletons may appear selectively depending on work direction.

The work distance factor also showed significant differences in several muscles. Significance was confirmed for the left and right AD, left ES, right VL, and BB (*p* < 0.05), and muscle activity was generally higher for the far distance. This reflects increased moment load as the lever arm of the arm lengthens. However, with Airframe use, this increase was mitigated, and in some conditions, muscle activity remained lower than or similar to No Exo even at the far distance.

Wearing the Airframe significantly reduced muscle activity in major shoulder and upper-arm muscles, demonstrating an upper-limb load-reduction effect. In contrast, increased activation of trunk muscles was observed, indicating that reductions in upper-limb load may shift stabilization demands toward the core. These findings suggest that the effects of exoskeletons do not result in uniform changes solely from device use; rather, the outcomes vary depending on the combination of exoskeleton condition, work direction, and distance. Therefore, when designing and applying exoskeletons, it is important to consider not only the efficiency of upper-limb assistance but also the potential increase in trunk stabilization demands.

### 3.2. Evaluation of Postural Stability

**Figure 4:**
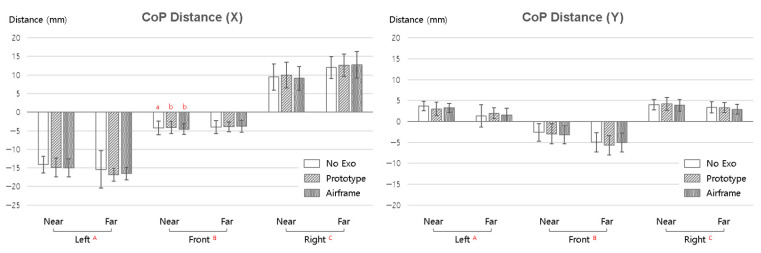
Mean CoP travel distance (X- and Y-axes) by exoskeleton condition, work direction, and distance (near/far). (Capital letters A, B, and C indicate statistically homogeneous groups identified by the SNK post hoc test; groups sharing the same letter do not differ significantly. Lowercase letters a and b denote significant simple effect differences.).

Figure 4 shows the CoP travel distance according to the exoskeleton condition (No Exo, Prototype, and Airframe), work direction (left, front, and right), and work distance (near and far). All post hoc comparisons used the SNK method. MANOVA results showed no significant differences by exoskeleton condition (*p* > 0.05), while work direction and distance were statistically significant on both axes (X and Y) (*p* < 0.05).

For CoP Distance-X, main effects of direction and distance were observed. In terms of absolute values, the leftward work region showed the largest magnitude of CoP travel, interpreted as greater trunk rotation and lateral lean compensation as the target deviates from the midline. Far distance significantly increased the absolute value of CoP travel compared to near, indicating that the further the arm and trunk are extended, the greater the lateral weight shift required to maintain balance. All three exoskeleton conditions (No Exo, Prototype, and Airframe) showed similar patterns, and wearing the Airframe did not produce additional suppression of center of mass movement or induce instability.

For CoP Distance-Y, direction and distance were also significant, but the absolute magnitude of travel was smaller than on the X-axis. Overall, the far distance slightly increased the magnitude of anterior–posterior CoP movement, which can be seen as the result of forward trunk inclination and center of mass shift. Differences by exoskeleton condition were not significant, and the pattern of anterior–posterior center of mass movement was consistently maintained even with Airframe use.

**Figure 5:**
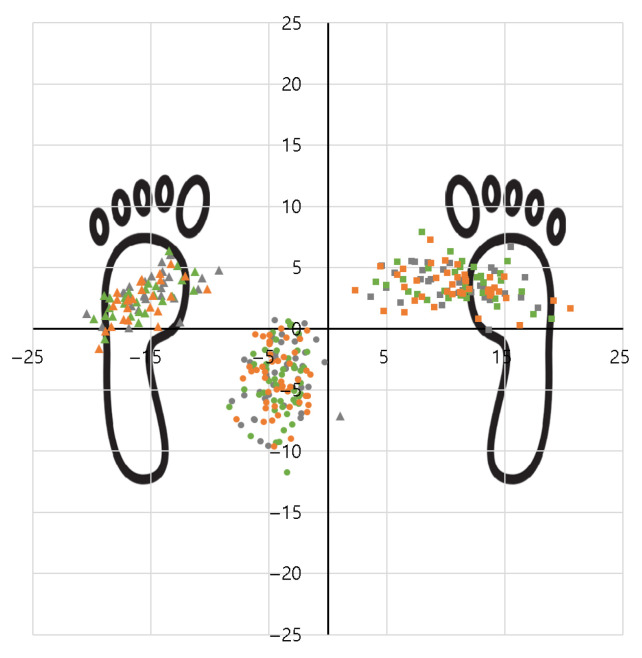
Spatial distribution of CoP trajectories overlaid on foot outlines for each work direction (left: triangles; front: circles; right: squares) and exoskeleton condition (gray: No Exo; green: Prototype; orange: Airframe).

Figure 5 provides a visual representation of the spatial distribution of CoP. The background displays foot morphology based on the 50th percentile of Korean males aged 20–29 years, with a foot straight length of 255.0 mm and foot width of 98.1 mm [26]. The triangular marker indicates leftward tasks, the circular marker indicates front tasks, and the square marker indicates rightward tasks; colors distinguish exoskeleton conditions (gray—No Exo; green—Prototype, red—Airframe). Colors are unified by exoskeleton condition regardless of distance.

As shown in the figure, CoP distributions are clearly separated within the foot morphology by work direction, with a tendency for the center to move into the left foot region during leftward tasks. In contrast, front tasks produce CoP distributions clustered around the midfoot, and rightward tasks shift CoP toward the right forefoot. These left–right symmetric patterns are consistent with the numerical analysis of CoP Distance-X and visually demonstrate that the trunk maintains stable balance while rotating and tilting asymmetrically according to the work direction.

By exoskeleton condition, the centers and ranges of the color distributions almost overlap; even when wearing the Airframe, CoP clusters did not spread or bias separately. This means that wearing the exoskeleton relieved upper-limb load while maintaining the user’s existing postural control strategy, without adversely affecting postural stability.

The CoP analysis shows that the exoskeleton acted as an assist device that reduces muscle load while preserving balance control. In particular, the absolute values of center of mass travel were largest for the left direction and far distance, confirming that postural compensation increases as task asymmetry increases. The Airframe accommodates such compensatory trunk responses without inducing instability and possesses structural stability.

### 3.3. Task Completion Time

**Figure 6:**
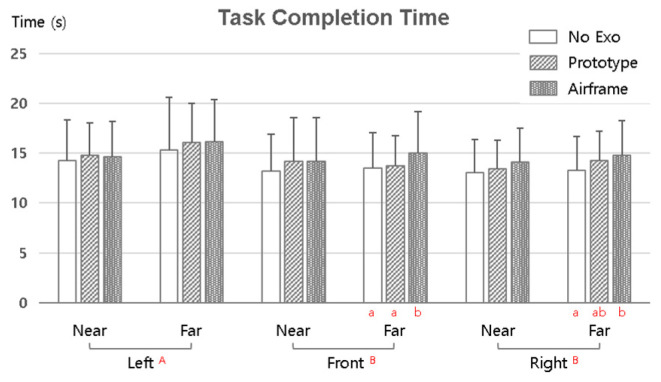
Mean task completion time by exoskeleton condition, work direction (left/front/right), and distance (near/far). (Capital letters A, B, and C indicate statistically homogeneous groups identified by the SNK post hoc test; groups sharing the same letter do not differ significantly. Lowercase letters a and b denote significant simple effect differences).

Figure 6 shows the mean task completion time as a function of the exoskeleton condition (No Exo, Prototype, and Airframe), work direction (left, front, and right), and work distance (near and far). Post hoc analyses used the SNK method. MANOVA results indicated no statistically significant differences by exoskeleton condition or distance (*p* > 0.05), but a significant main effect of direction (*p* < 0.05). That is, task direction directly affected completion time more than exoskeleton use or distance.

Overall, mean completion times ranged approximately from 13 to 17 s for all conditions, and no significant increases or decreases were observed with Airframe use. This implies that the assist structure of the Airframe did not reduce overall task efficiency even while alleviating upper-limb physical burden. The Prototype also showed completion speeds similar to No Exo, with limited operational delay or discomfort due to wearing the device.

In contrast, clear differences appeared for the direction factor. The SNK post hoc test showed that completion time was longest for leftward tasks (A) and tended to decrease for front (B) and right (C). This is interpreted as the result of longer posture transitions and arm movement paths as the task target deviates from the body midline. Some simple-effect test results (a and b) demonstrated statistically significant differences between leftward and rightward tasks.

Wearing the exoskeleton itself did not adversely affect task efficiency, and task direction acted as the principal determinant of completion time. That is, wearing the Airframe reduced upper-limb load while preserving functional efficiency such as maintaining task speed.

### 3.4. Subjective Measures

**Figure 7:**
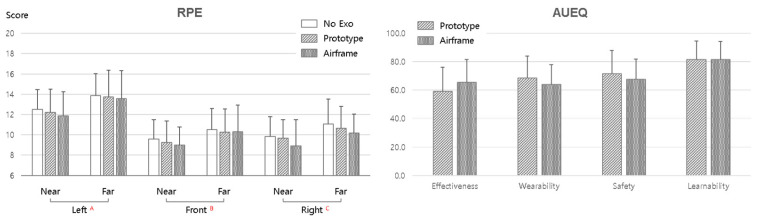
Subjective evaluation results. (**Left**) Mean whole-body perceived exertion (RPE) by exoskeleton condition, work direction, and distance; (**Right**) AUEQ scores. (Capital letters A, B, and C indicate statistically homogeneous groups identified by the SNK post hoc test.).

Figure 7 shows the results of subjective load and usability assessments. Subjective load was evaluated using RPE and RPD, and usability was measured with the AUEQ. Figure 7 presents the results for whole-body RPE. MANOVA results indicated no significant differences by exoskeleton condition (*p* > 0.05), but significant main effects of direction and distance (*p* < 0.05). Overall, RPE was higher for the far distance than for the near distance, interpreted as an increase in physical fatigue due to the greater demands of reaching the arm and trunk further. RPE was also highest for the left direction (A) and tended to decrease toward the right direction (C). This suggests that physiological load required for posture control and balance maintenance increases as the task target deviates from the body center.

There were no statistically significant differences among exoskeleton conditions, but the mean RPE tended to be slightly lower with Airframe. This suggests the possibility that Airframe reduced upper-limb muscular load and thereby alleviated perceived fatigue. In contrast, the Prototype showed levels similar to No Exo, and the device’s mechanical assistance did not clearly translate to perceived fatigue.

The same tendency appeared in RPD assessments. RPD was measured for nine regions (neck, shoulder, upper back, elbow, wrist, lower back, hip/thigh, knee, and ankle/foot), and direction and distance factors were significant for most regions (*p* < 0.05). In particular, pronounced direction effects appeared in the shoulder, lower back, and upper back, and discomfort increased for the far distance. Only the wrist was not statistically significant for the distance factor (*p* > 0.05), which is interpreted as due to the manipulation of work distance not directly affecting wrist rotation/flexion load.

Figure 7 presents the AUEQ results. The scale comprises four sub-factors—Effectiveness, Wearability, Safety, and Learnability—and was used to compare the Prototype and Airframe. Analysis showed a significant difference only for the Effectiveness factor (*p* < 0.05), with the Airframe scoring higher than the Prototype. This suggests that the Airframe possesses a structural design that more clearly communicates actual assistive effects to the user. In contrast, no significant differences appeared for Wearability, Safety, or Learnability, indicating that both devices were evaluated at similar levels for donning comfort, safety, and ease of learning.

The subjective evaluation results showed the following characteristics: (1) in RPE and RPD, work direction and distance acted as the principal factors for fatigue and discomfort, not exoskeleton wearing per se; (2) with Airframe, a tendency toward lower average fatigue was observed; and (3) in AUEQ, only Effectiveness improved significantly, reflecting the Airframe’s substantive assistive effect.

## 4. Discussion

### 4.1. Analysis of Exoskeleton Effects in Agricultural Tasks

This study conducted an integrated analysis of how an upper-limb assist exoskeleton affects muscle activity, postural stability, performance efficiency, fatigue, and usability perception during repetitive and asymmetric postures (in particular, variations in direction and distance) in agricultural work environments. As a result, the Airframe-type exoskeleton reduced muscular load while maintaining task performance efficiency and postural stability.

First, the physiological indicator analysis clearly showed the physical advantage of wearing the exoskeleton. With Airframe wear, muscle activity of the UT and AD decreased significantly, with mean reductions of about 25–35 percent. This is interpreted as due to the Airframe’s spring mechanism effectively sharing the torque generated during arm elevation, thus reducing shoulder and upper-arm muscular load. These results suggest potential mitigation of cumulative fatigue during agricultural tasks that frequently require prolonged arm elevation (e.g., pruning, harvesting, and spraying).

Meanwhile, erector spinae showed a tendency for increased activation when wearing the exoskeleton. This is interpreted as compensatory core activation that raises trunk stabilization demands when upper-limb load is reduced, indicating that exoskeleton wear not only reduces the upper-limb burden but also induces new forms of trunk balance control. Such results suggest that, in environments like agricultural work—where unbalanced postures and repetitive trunk movements are common—exoskeleton design should consider postural synergy along with upper-limb assistance.

In the CoP analysis, direction and distance significantly affected CoP travel, but there were no differences among exoskeleton conditions. In other words, the Airframe reduced upper-limb load without compromising stability of the body center. In particular, the absolute values of center of mass travel were largest for the left direction and far distance, indicating increases in trunk balance control demands as task asymmetry increases. Nonetheless, no instability due to exoskeleton wear was observed, confirming that the Airframe functions as a safe assist mechanism that preserves postural stability.

Task completion time was influenced more by direction than by exoskeleton wear or distance. Completion time was longer for the left direction and tended to shorten for the front and right directions, interpreted as longer motion transitions and arm movement paths in eccentric postures that deviate from the midline. However, no increase in completion time or reduction in efficiency due to the Airframe wear was observed, confirming that wearing the assist device does not hinder productivity.

In subjective fatigue and discomfort assessments, both direction and distance were significant; whole-body fatigue and local discomfort increased for the far distance and left direction. While there were no statistically significant differences among exoskeleton conditions, the average fatigue tended to be lower with the Airframe, suggesting that, even if reductions in muscle activity do not fully translate to relief of perceived fatigue, cumulative fatigue mitigation can be expected in long-duration repetitive tasks.

Finally, in the AUEQ results, the Airframe recorded a significantly higher score than the Prototype only for the Effectiveness factor. This means that users perceived the Airframe as a device that actually helps in work tasks, supporting the congruence between physiological efficiency (EMG reduction) and perceived effectiveness. There were no significant differences for Wearability, Safety, or Learnability, which indicates that while both devices secured basic donning stability, the Airframe placed emphasis on functional efficiency in its design.

The Airframe-type exoskeleton showed a balanced performance profile across four aspects: reduction in physical load, maintenance of postural stability, preservation of task efficiency, and securing of cognitive acceptance. In environments where long-duration asymmetric movements are repeated, such as agricultural work, these results suggest the potential use of exoskeletons as effective assist devices to prevent muscle fatigue and enhance task sustainability. Moreover, inconsistencies between subjective fatigue and muscle activity highlight the importance of considering physical–cognitive interaction in design, beyond simple strength assistance.

### 4.2. Characteristics and Significance of Each Metric

This study verified the effects of wearing an exoskeleton from multiple perspectives using four indicators—muscle activity, postural stability, subjective fatigue and discomfort, and AUEQ. Each indicator represents a different evaluation domain and complements the physical and cognitive effects of exoskeletons.

First, EMG was evaluated as the most representative physical indicator. By quantitatively measuring muscle contraction levels during tasks, EMG directly shows whether an exoskeleton actually reduces load. In this study, %MVC decreased significantly in the main shoulder and upper-arm muscles with Airframe wear, clearly proving the device’s mechanical assistance efficiency. However, EMG does not necessarily coincide with perceived fatigue or usability, meaning that reductions in physiological burden do not always lead to relief of perceived fatigue—because cognitive effort and sensory factors act independently during tasks. Even so, EMG is a key indicator for evaluating the mechanical efficiency of exoskeletons and is likely to be established as a standard evaluation index in industry.

Second, the CoP indicator functioned as a useful supplementary indicator for evaluating whole-body stability. CoP analysis allows indirect assessment of postural stability and trunk control strategy through center of mass movement. In this study, direction and distance clearly affected CoP movement, and there were no differences among exoskeleton conditions. This means that the Airframe did not hinder balance control or change existing postural control patterns. However, in upper-limb-centered tasks, changes in CoP can be limited, which constrains detection of subtle differences in balance control. Therefore, CoP has considerable meaning as a supplementary indicator of the indirect effect of exoskeletons on body center movement and postural strategy.

Third, RPE and RPD were used as subjective indicators reflecting user-perceived fatigue and discomfort. Although they do not directly correlate with physiological indicators, they are effective for monitoring acceptance and cumulative fatigue risk from the user’s perspective. In this study, direction and distance were significant in RPE and most RPD regions, and a tendency for lower average fatigue with the Airframe was observed. However, subjective evaluations are subject to large individual differences and cognitive–emotional influences, which creates limitations in quantitative comparisons with objective loads. Even so, RPE/RPD holds importance for predicting user compliance and the risk of cumulative fatigue in long-term wear.

Fourth, AUEQ is a scale that comprehensively evaluates usability and effectiveness and enables integrated interpretation of technology–user interaction. In this study, the Airframe recorded significantly higher scores than the Prototype on Effectiveness, indicating that the actual assistive effects of the exoskeleton were perceived as efficiency by users. There were no significant differences in Wearability, Safety, or Learnability, which can be seen as the result of both devices securing basic donning comfort and safety. Although AUEQ is limited to short-term usability assessments, it has significance as a key tool for validating user experience-based design.

In sum, the multimodal assessment adopted in this study allowed for a three-dimensional interpretation of exoskeleton effects from physiological, behavioral, and cognitive perspectives rather than a single viewpoint (Table 1). EMG represents mechanical efficiency, CoP represents changes in postural stability, RPE/RPD represent perceived fatigue and compliance, and AUEQ represents usability-based cognitive acceptance. Such complementary analyses among indicators can serve as foundational material for the advancement of future evaluation systems for exoskeletons and provide practical grounds for assessing real-world suitability in industry.

### 4.3. Interrelationship Among Evaluation Metrics

To understand exoskeleton effects in a multifaceted manner, it is necessary to interpret physiological, postural, subjective, and cognitive responses in an integrated way rather than relying on a single indicator. The four indicators used in this study—EMG, CoP, RPE/RPD, and AUEQ—each have independent meanings but form complementary relationships and comprise the overall context of exoskeleton effects.

EMG fundamentally plays the role of a core reference measure that supports the interpretation of other indicators. The finding that muscle activity in shoulder and upper-arm muscles decreased significantly with Airframe wear provides the underlying physiological basis for the changes observed in CoP, RPE, and AUEQ. For example, reductions in EMG appeared consistent with maintenance of CoP excursion (i.e., no deterioration in postural stability) and a slight tendency toward decreased RPE. This suggests a chain of bio-behavioral–cognitive responses: reduction in muscular load, maintenance of postural stability, and relief of perceived fatigue.

However, EMG alone cannot represent the overall effect of exoskeletons. While EMG shows “how much less effort was used,” CoP explains “what postural strategy was used to maintain balance,” RPE/RPD explain “how fatiguing the change felt,” and AUEQ explains “how the user perceived the change.”

Therefore, while EMG provides a foundation as the central indicator of physical load, parallel interpretation with CoP and subjective indicators is essential to explain the direction of psychological acceptance or changes in postural compensation. For example, the small increases in trunk muscle activity observed with the Prototype likely reflect natural variations in whole-body stabilization strategies rather than systematic effects of the device, as no significant differences in erector spinae activation were found between the Prototype and the Airframe.

Synthesizing the complementary relationships among indicators yields the following. In the relationship between EMG and CoP, reductions in muscular load do not necessarily lead to improved stability. In some conditions, even if upper-limb muscle activity decreases, compensatory increases in trunk muscle activity (erector spinae) may appear, indicating that reductions in EMG may inversely relate to CoP stability. That is, mechanical assistance that reduces the upper-limb burden can increase the burden of trunk posture control, and simultaneous interpretation of the two indicators can more clearly identify the indirect effects of exoskeleton wear on overall body stability.

In the relationship between EMG and RPE/RPD, moderate correlations (about 0.4–0.6) are generally reported, and similar tendencies were observed in this study. Although reductions in muscle activity with the Airframe tended to lead to relief of perceived fatigue, the magnitude was limited. This shows that, even if muscular physical load is reduced, the fatigue perceived by the subject may not decrease to the same extent. These results suggest that reductions in muscular load do not automatically mean improvements in fatigue perception and highlight the need to evaluate cumulative fatigue in long-term wear or repetitive work situations.

Finally, examining the relationship between EMG and AUEQ, the Effectiveness subscale is directly related to the efficiency of mechanical assistance and may show a positive correlation with EMG. In fact, the Airframe showed high Effectiveness scores along with reductions in EMG, supporting this consistency. However, Wearability, Safety, and Learnability are primarily determined by affective factors, such as donning comfort, stability perception, and ease of adaptation, and thus have low direct correlations with physiological indicators. Therefore, the overall evaluation of exoskeletons should not rely on a single indicator but should interpret physical load indicators and cognitive/psychological indicators (AUEQ and RPE/RPD) in a mutually complementary manner.

Taken together, EMG still plays a central reference role as the most reliable physical indicator, but combining it with CoP, RPE/RPD, and AUEQ maximizes interpretive completeness. In particular, the triple combination of EMG–CoP–RPE can function as a minimal triad to explain the link among physiological, behavioral, and subjective responses. Adding AUEQ extends interpretation to a cognitive layer that connects technical performance with user experience.

Therefore, future studies can simultaneously verify the real-world applicability and ergonomic utility of exoskeletons by building a multilayer integrated assessment framework that combines CoP and AUEQ with physiological indicators centered on EMG.

## 5. Conclusions

This study examined the effects of an upper-limb assist exoskeleton on muscle activity, postural stability, task efficiency, fatigue perception, and usability during repetitive and asymmetric agricultural movements. Increases in ES activation of exoskeleton robots suggested compensatory trunk involvement, but CoP analysis confirmed that balance control was not adversely affected. Subjective results showed slightly lower fatigue and higher perceived effectiveness for the Airframe compared with the Prototype.

This experiment involved a limited number of participants in controlled settings and did not address long-term use, lower-limb locomotion, or complex multi-stage agricultural tasks. Short-term subjective ratings (RPE and AUEQ) also limit interpretation of learning effects or long-term acceptance. Individual differences in anthropometry and device fit may have influenced the results.

Despite these limitations, this study offers meaningful academic and industrial insights. It integrates physiological, behavioral, and cognitive responses to propose a multidimensional framework (EMG–CoP–RPE/RPD–AUEQ) for evaluating agricultural exoskeletons. The proposed evaluation approach provides a foundation for standardized exoskeleton assessment and may contribute to improving both agricultural productivity and worker health amid increasing labor shortages.

## Figures and Tables

**Table 1:** Characteristics, interpretive limitations, and significance of the evaluation metrics used for exoskeleton assessment.

Metric	Representativeness	Interpretation and Limitations	Significance
EMG	High	A core indicator that quantifies physical load; does not directly reflect perceived fatigue.	Essential for evaluating an exoskeleton’s mechanical efficiency; potential as a future standard indicator.
CoP	Moderate	Useful for evaluating whole-body stability, but with limited change ranges in upper-limb-centered tasks.	A supplementary indicator for analyzing changes in postural strategy.
RPE/RPD	Low	Large individual differences and low correlation with physiological indicators.	For monitoring compliance and cumulative fatigue.
AUEQ	Moderate to high	Integrates usability, efficiency, and safety, but limited to short-term evaluation.	A key tool for validating designs based on user experience.

## Data Availability

All data included in this study are available upon request by contacting the corresponding author.

## Abbreviations

The following abbreviations are used in this manuscript:

AD	Anterior Deltoid
AUEQ	Agricultural Exoskeleton Usability Evaluation Questionnaire
BB	Biceps Brachii
CoP	Center of Pressure
EMG	Electromyography
ES	Erector Spinae
MVC	Maximum Voluntary Contraction
RPD	Rating of Perceived Discomfort
RPE	Rating of Perceived Exertion
SNK	Student–Newman–Keuls
UT	Upper Trapezius
VL	Vastus Lateralis
